# A malaria risk map of Kinshasa, Democratic Republic of Congo

**DOI:** 10.1186/s12936-015-1074-8

**Published:** 2016-01-13

**Authors:** Giovanfrancesco Ferrari, Henry M. Ntuku, Sandro Schmidlin, Eric Diboulo, Antoinette K. Tshefu, Christian Lengeler

**Affiliations:** Swiss Tropical and Public Health Institute, Basel, Switzerland; University of Basel, Basel, Switzerland; Kinshasa School of Public Health, Kinshasa, Democratic Republic of the Congo

**Keywords:** Malaria, Anaemia, Mosquito nets, DRC, Democratic Republic of Congo, Kinshasa

## Abstract

**Background:**

In Kinshasa, malaria remains a major public health problem but its spatial epidemiology has not been assessed for decades now. The city’s growth and transformation, as well as recent control measures, call for an update. To identify highly exposed communities and areas where control measures are less critically needed, detailed risk maps are required to target control and optimize resource allocation.

**Methods:**

In 2009 (end of the dry season) and 2011 (end of the rainy season), two cross-sectional surveys were conducted in Kinshasa to determine malaria prevalence, anaemia, history of fever, bed net ownership and use among children 6–59 months. Geo-referenced data for key parameters were mapped at the level of the health area (HA) by means of a geographic information system (GIS).

**Results:**

Among 7517 children aged 6–59 months from 33 health zones (HZs), 6661 (3319 in 2009 and 3342 in 2011) were tested for both malaria (by Rapid Diagnostic Tests) and anaemia, and 856 (845 in 2009 and 11 in 2011) were tested for anaemia only. Fifteen HZs were sampled in 2009, 25 in 2011, with seven HZs sampled in both surveys. Mean prevalence for malaria and anaemia was 6.4 % (5.6–7.4) and 65.1 % (63.7–66.6) in 2009, and 17.0 % (15.7–18.3) and 64.2 % (62.6–65.9) in 2011. In two HZs sampled in both surveys, malaria prevalence was 14.1 % and 26.8 % in Selembao (peri-urban), in the 2009 dry season and 2011 rainy season respectively, and it was 1.0 % and 0.8 % in Ngiri Ngiri (urban). History of fever during the preceding two weeks was 13.2 % (12.5–14.3) and 22.3 % (20.8–23.4) in 2009 and 2011. Household ownership of at least one insecticide-treated net (ITN) was 78.7 % (77.4–80.0) and 65.0 % (63.7–66.3) at both time points, while use was 57.7 % (56.0–59.9) and 45.0 % (43.6–46.8), respectively.

**Conclusions:**

This study presents the first malaria risk map of Kinshasa, a mega city of roughly 10 million inhabitants and located in a highly endemic malaria zone. Prevalence of malaria, anaemia and reported fever was lower in urban areas, whereas low coverage of ITN and sub-optimal net use were frequent in peri-urban areas.

## Background

Malaria is the leading cause of morbidity and death in children under 5 years in the Democratic Republic of Congo (DRC), accounting for an estimated 40 % of outpatient visits and 40 % of overall mortality [1]. Malaria is also a major public health issue in the capital city Kinshasa; an issue that has been studied since colonial times [2]. After Cairo and Lagos, Kinshasa is Africa’s third largest city, with an estimated population of more than 10 million [3]. In 1979–1980, the average malaria parasite rate in a representative sample of children was 33 % [2]. Around the same time, malaria admissions comprised 29.5 % of consultations in 1983, then 38.2 % in 1985–1986 [4]. In 1986–1987, the mean prevalence rate of malaria in six districts of Kinshasa was 50 %, with a higher prevalence in the peripheral districts [5]. This reflected the distribution pattern of the main vector *Anopheles gambiae,* which was less present in the city centre than in the periphery [6, 7]. The latest study in 2000 confirmed the general prevalence distribution pattern, with lower prevalence in the city centre (parasite rate 4 %) than in peri-urban areas (46 %) [8].

A first insecticide-treated net (ITN) distribution campaign in 2007 achieved a 15.9 % rate of ITN ownership and a 12.6 % rate of use among children under five [9]. In 2008, the World Bank financed the acquisition and distribution of two million ITNs in Kinshasa through the PURUS project (*Programme d’Urgence de Réhabilitation Urbaine et Sociale*). The National Malaria Control Programme (NMCP), along with technical and logistic support from Population Services International (PSI), distributed two ITNs per household. Eight months after that distribution, the Kinshasa School of Public Health (KSPH) conducted a survey on basic malaria indicators to assess the impact of the intervention in 15 health zones (HZs) of the city. In 2011, the Swiss Tropical and Public Health Institute (Swiss TPH), in collaboration with the KSPH, conducted a second survey to evaluate the coverage and use of key malaria indicators, prevalence of malaria by Rapid Diagnostic Test (RDT), anaemia and fever in the 23 HZs excluded from the 2009 survey. Kinshasa has expanded very rapidly in the past 20 years, thus updating and consolidating these data was urgently required for general malaria control purposes and for planning specific further research projects. Using geo-referenced prevalence data, this study aimed to generate the first map of malaria risk among children 6–59 months in Greater Kinshasa, down to the lowest level of the health system in DRC, the health area (HA). These maps will enable researchers and implementers to identify HZs of high priority for malaria control in Kinshasa.

## Methods

### Study area

The study was conducted in Kinshasa, the capital of the DRC. The city is located along the southern bank of the Congo River, directly opposite the city of Brazzaville, capital of the Republic of the Congo. The climate is hot and humid (AW4 according to the Koppen classification), with a rainy season lasting from October to May [10]. Characterized in the north by the Pool Malebo and by a marshy area in the north-east along the river Congo, Kinshasa extends across a plain delimited to the south by hills with heights varying between 350 and 750 meters. The plain is crossed by three rivers (Ndjili, Nsele and Mai-Ndombe) and many smaller streams [11, 12]. The northern and central parts of the city include the old colonial neighbourhoods (*ville*), some of which represent the most industrialized and commercial areas. To the south lies the *cité*, consisting of more recent, large, residential districts. The land use pattern is heterogeneous, with densely populated areas separated by large semi-rural areas where urban agriculture is practiced. The most heavily inhabited area of Kinshasa covers 583 square km [13], of which 80 % is actually semi-rural.

Administratively, Kinshasa has the status of a province, divided into four districts, which are further divided into 24 *communes* (municipalities). The organization of the health system differs from the administrative system and comprises six health districts, divided into 35 health zones (HZs). These represent the primary operational units of the health system in DRC. An HZ usually covers a population of 100,000–150,000 inhabitants in rural areas and 200,000–250,000 in urban centres. They include a general referral hospital, some health centres and a dozen lower-level health facilities. Each HZ is further divided into 15 health areas (HAs), on average, which represent the lowest level of the health system. Each HA is clearly delimited and defined by the Ministry of Health and usually includes a population of 10,000–15,000 inhabitants. In Kinshasa Province, the three most eastern HZs are completely rural in nature, while the remaining 32 HZs are semi-rural or urbanized [14]. The study area only consisted of the 32 non-rural HZs because of the practical issues involved in including the three eastern HZs. Details of the sampled HZs can be found in Table 1.

**Table 1:** List of health zones in Greater Kinshasa surveyed in 2009 (KSPH/NMCP) and 2011 (KSPH/Swiss TPH) and corresponding populations

Health zone	Environment	Population	Year survey
Bandalungwa	Urban	147.252	2011
Barumbu	Urban	115.780	2011
Binza Meteo	Urban	325.446	2009^a^/2011
Binza Ozone	Urban	317.731	2011
Biyela	Urban	174.232	2009^a^/2011
Bumbu	Urban	316.188	2009
Gombe	Urban	22.732	2011
Kalamu I	Urban	112.915	2011
Kalamu II	Urban	100.782	2011
Kasa-Vubu	Urban	102.856	2009
Kikimi	Urban	198.997	2011
Kimbanseke	Urban	217.772	2011
Kingabwa	Urban	162.323	2009
Kingasani	Urban	171.538	2011
Kinshasa	Urban	135.665	2011
Kintambo	Urban	81.026	2011
Kisenso	Urban–rural	335.265	2009
Kokolo	Urban	336.086	2009
Lemba	Urban	249.292	2009^a^/2011
Limete	Urban	145.331	2009^a^/2011
Lingwala	Urban	66.595	2011
Makala	Urban	238.088	2011
Maluku I	Urban	149.040	Excluded
Maluku II	Rural	54.158	2009
Masina I	Urban	258.687	2011
Masina II	Urban	214.401	2009^a^/2011
Matete	Urban	223.248	2009
Mont Ngafula I	Urban–rural	196.810	2011
Mont Ngafula II	Urban–rural	111.921	2011
N’djili	Urban	249.310	2009
Ngaba	Urban	140.861	2011
Ngiri Ngiri^b^	Urban	125.634	2009/2011
Nsele	Rural	387.486	Excluded
Police	Urban	93.910	2011
Selembao^b^	Urban	269.498	2009/2011

^a^Surveyed for malaria preventive indicators and prevalence of anaemia

^b^Surveyed for malaria prevalence in both years and for all age groups in 2011

### Study design and sampling procedure

Two cross-sectional household surveys were conducted. The first survey was carried out at the end of the dry season between mid-September and end of October 2009 in 15 HZs, eight months after the first large ITN distribution campaign. The second survey was conducted at the end of the rainy season from mid-April to early June 2011 and covered 25 non-rural HZs. Seven HZs were sampled in both studies, including five HZs for which malaria prevalence was not measured in 2009 and two HZs for which prevalence was measured previously in 2009. The detailed list of HZs surveyed in 2009 and 2011 is presented in Table 1. For both surveys, a multi-stage cluster sampling design was adopted to select households for inclusion, using the HZ as a primary sampling stage and the HA as a secondary sampling stage.

### 2009 survey

Fifteen HZs were selected using a probability proportional to size (PPS) sampling method, so that more populated HZs had a higher probability of being selected. Of these 15 HZs, 10 were selected by simple random sampling for the determination of malaria by rapid diagnostic test (RDT). In the remaining five HZs, only haemoglobin (Hb) was measured and malaria preventive measures were investigated using a pre-tested, structured questionnaire. In each HZ, data collection took place in half of the HAs, selected again with PPS. In case of an odd number of HAs per HZ, (n + 1)/2 HAs were selected. In a third stage, a list of all streets with their approximate population number was obtained for each selected HA. Streets with fewer than 200 inhabitants were excluded and three streets were selected by simple random sampling. Households with at least one child aged 6–59 months were listed by community health workers (CHW) for each of the three streets. From this list, 25 eligible households were randomly selected, proportional to the size of each street. The target sample size of 325 children was calculated based on an estimated prevalence of anaemia of 69.2 % in children aged 6–59 months. Assuming 1.3 children aged 6–59 months per household, a sample size of 260 households was set as the target per health zone [9].

## 2011 survey

From mid-April to early June 2011, the remaining 23 HZs were sampled, including the five HZs for which malaria prevalence had not been measured in the 2009 survey. In all 23 HZs, a questionnaire was administered to households and malaria parasite prevalence and the Hb concentration were measured in children aged 6–59 months. Two additional HZs already investigated in 2009 were re-sampled in 2011 among children 6–59 months for both malaria and anaemia. To obtain the epidemiological age profile for all age groups in these latter HZs only, individuals older than five were also included. In all, 25 HZs were sampled in the 2011 survey. The primary outcome measure was documented malaria in study children, as measured by RDT. The sample size was calculated based on the prevalence estimate for 2009 survey (6.4 %) during the dry season, and increased to 10 % to take into account the seasonal variation. In each HZ, the aim was to measure children’s malaria with a precision of ±8 absolute percent. The sample size calculation indicated the need for 55 children in each HZ. With a design effect accounting for clustering of two, this number increased to 110. With an average 1.3 children under 5 years in households in Kinshasa, 87 households needed to be selected [9]. To account for losses in the study process, we aimed for 100 households in each of the 25 HZs. Hence, the total number of households sampled in Kinshasa in 2011 was 2500, including 3250 children aged 6–59 months. HA and household selection followed the same methodology applied in the 2009 survey (described above). An average of 25 households was set per HA.

### Data collection

#### Household survey questionnaire

In 2009, survey data were collected using a paper-based questionnaire. In 2011, survey data were collected using smartphone technology. For the 2011 survey, a validated electronic semi-quantitative questionnaire was developed on an HTC smartphone running Google’s Android operating system. Eight teams of three field workers (one interviewer, one laboratory technician, one community liaison person) were trained in using the electronic questionnaire, in general interviewing skills and in administering informed consent during simulated interviews sessions. Each of the eight teams visited, on average, 25 households per day in each selected HA. The 2011 questionnaire was a simplified version of the one used in 2009, which was adapted from the standard Malaria Indicator Survey Household Questionnaire from the Roll Back Malaria Partnership. All questions retained from the 2009 survey form were kept as they were in 2011 to ensure comparability between both surveys. The questionnaire was developed in French with oral translation into Lingala (the second *lingua franca* in Kinshasa) and field tested prior to the survey.

Prior to administering the questionnaire, a signed informed consent form was obtained from the head of the household or his/her representative. Participation was entirely voluntary. Respondents were asked about demographic information of usual residents, educational level, factors indicating the household’s socio-economic status, household construction material, presence and type of mosquito bed net (verified by direct observation), use of mosquito bed net and ITN in the night prior to the survey, history of fever (past 2 weeks), whether fever was present on the day of the survey and health seeking behaviour in case of a fever episode. During the 2011 survey, the coordinates (longitude and latitude) of all investigated households were recorded on-site using the integrated Global Positioning System (GPS) of the data collection devices. Households were revisited if no one was available for interview on the first attempt; if no one was available after two attempts, the interviewer continued to the next randomly selected household on the list until the desired number of households was obtained.

#### Blood testing

For each selected participant who gave signed informed consent, the same laboratory procedures as in 2009 were adopted during the 2011 survey. They included measuring axillary temperature with a digital thermometer, collecting peripheral blood by standard finger prick to test for malaria parasites with an RDT for *Plasmodium falciparum*-specific histidine rich protein 2 (HRP2) and other *Plasmodium species* (Pan pLDH for *Plasmodium vivax, Plasmodium malariae* and *Plasmodium ovale*) (Paracheck pf in 2009 and SD Bioline Malaria Antigen P.f/Pan in 2011) and assessing Hb level using a blood haemoglobin photometer (HemoCue 201 plus, Ängelholm, Sweden). In two HZs in 2011, Selembao and Ngiri Ngiri, individuals of all ages (not only children) were surveyed. RDTs were used for on–site diagnosis of malaria and treatment with artesunate-amodiaquine, the official first-line malaria treatment at the time of the survey, was offered as needed. The HemoCue was validated by running a weekly high and low Hb liquid control (HemoCue–HemoTrol).

### Statistical analysis

To ensure consistency and integrity of data collected during the 2009 survey, all paper forms were rechecked by team supervisors in the field at the end of each day. Incomplete entries were sent back to be filled the next day. Questionnaires were first checked for completeness, and the information was manually coded and entered using EpiData and crosschecked using EpiInfo (v. 6.04). Statistical analyses were performed using SPSS software for Windows (version 16.0), NCSS, and STATA (version 10).

Data collection devices used in the 2011 survey (HTC phones) were equipped with Open Data Kit (ODK) software (University of Washington and Google Foundation) to allow for data entry in the field. ODK programming also allowed for systematic range and consistency checks. Data in xml format were downloaded every evening from the HTC smartphones and then converted on the ODK Aggregate Server into tabular format (ODK aggregate).

Statistical analyses were performed using Stata version 12.1 (Stata Corp, College Station, Tx, USA). Analysis and mapping for the 2011 survey were based on geo-referenced prevalence data at the level of the HA. Since households could not be georeferenced in 2009, HA spatial coordinates were assigned to the HA’s mean malaria prevalence. Maps were produced using ArcGIS version 10.0 (Environmental Systems Research Institute Inc. Redlands, USA). A centroid for every HA was first generated (for 2009 the centroid was generated at the centre of the HZ, since GPS coordinates of the households were not collected). The standardized prevalence data were then assigned to the centroids of the surveyed health zone. The next step involved using the IDW interpolation to get prevalence estimates at un-surveyed HZs. Lastly, the interpolated prevalence estimates were extracted using the centroids (points data) of the HZs. These estimates were subsequently used to map out the prevalence at HZs level (polygons data). Boundaries (shape files) were initially available at the level of the HZ only, from the Health Monitoring Information System Unit of the Ministry of Health (MoH). By using images developed by the Japan International Cooperation Agency (JICA) and through collaborating with a team of experts from the *Institut géographique du Congo* (IGC), it was possible to develop shape files at the level of the HA. The most eastern rural HZs (Maluku I and II and Nsele) were excluded from the final map due to the great effort that drawing boundaries in remote HAs would have entailed. This was beyond the means and the scope of this study.

### Ethical consideration

For both surveys, ethical clearance was obtained from the Ethics Committee of the KSPH, at the University of Kinshasa. In addition, the 2011 survey received authorization from the ethical committees in Basel (Ethikkommission beider Basel, Basel-Stadt) as well as clearance from Swiss TPH’s internal research commission. Signed informed consent to participate was obtained from parents or guardians on behalf of the enrolled children or by the adult participants themselves. Precautions to minimize the risk of secondary infection during blood collection were taken. All tested participants with a positive RDT but no evidence of severe illness were diagnosed as having uncomplicated malaria and given a voucher for treatment, free of charge, as per the DRC national malaria treatment policy (artesunate-amodiaquine or artemether-lumefantrine), at the nearest health facility. Drugs were provided to the relevant facilities one day before the household visits started in the area, to ensure drug availability for treatment. Participants diagnosed with severe anaemia and those with severe illnesses were excluded from the study and immediately referred to the nearest health facility for diagnosis and management, as recommended by national guidelines.

## Results

### Characteristics of the study population

Household and individual characteristics of the study populations in 2009 and 2011 are shown in Table 2. A total of 3896 households distributed throughout 15 HZs were included in the 2009 survey, while 2,512 household in 25 HZs were sampled in 2011. The age distribution of individuals was similar between surveys, as were the proportions of men and women. Overall, 27,371 people were surveyed in 2009, including 12,761 men and 14,610 women. Of these, 47.1 % were under 15 years of age, while the percentage of children 6–59 months was 24 %. In addition, 302 pregnant women also participated. The 2011 survey included 15,005 people; 6770 men and 8235 women. Of these, 44.7 % were under 15 years of age, while the percentage of children 6–59 months was 24.9 %.

**Table 2:** Characteristics of study households and individuals in the 2009 and 2011 surveys, Kinshasa, Democratic Republic of Congo

	Survey 2009	Survey 2011
Household characteristics		
Number of households sampled	3896	2512
Mean (SD) household size	7.1	5.9 (2.1)
Individual characteristics		
Number of persons in sampled households	27,371	15,005
Median Age years (90 % central range)	–	17
Age groups		
<6 months (%)	–	0.9
6–59 months (%)	24.0	24.9
5–9 years (%)	13.1	10.9
10–14 years (%)	10.0	8.9
15–19 years (%)	9.2	8.1
≥20 years (%)	43.7	46.2
Proportion of females (%)	53.4	54.9

### Prevalence of *Plasmodium falciparum* by health zone

Table 3 gives the proportion of children 6–59 months who tested positive for malaria with RDT, by sampled HZs. A total of 3,319 children 6–59 months in 10 HZs were tested for malaria by RDT in the 2009 survey, whereas 3342 were tested in 25 HZs in 2011. Prevalence of confirmed malaria was 6.4 % (5.6–7.4) at the end of the 2009 dry season, ranging from 1.0 % (0.3–2.6) in Ngiri Ngiri (urban centre) to 14.1 % (10.6–18.2) in Selembao (peri-urban). At the end of the 2011 wet season, malaria prevalence was 17.0 % (15.7–18.3), ranging from 0.7 % (0.0–4.1) in Kinshasa and Lingwala (urban centre) to 46.0 % (37.1–55.1) in Biyela (peri-urban). *Plasmodium falciparum* accounted for 52 % (95 % CI 47.4–55.8) of infections in 2011 survey, non-falciparum infections for 0.3 % (95 % CI 0.0–1.3) while mixed infection (were not distinguished) prevalence was 48 % (95 % CI 43.9–52.3).

**Table 3:** Clinical outcomes, by health zone

Health zone	Malaria prevalence in children aged 6–59 months							Anaemia prevalence in children aged 6–59 months						Children <5 years with a fever episode in the 2 weeks before the survey			
	Survey 2009 (dry season)			Survey 2011 (wet season 2011)			Standardized prevalence	Survey 2009			Survey 2011			Survey 2009		Survey 2011	
	%	[95 % CI]	N	%	[95 % CI]	N	%	% severe (<7 g/dl)	% any (<11 g/dl)	N	% severe (<7 g/dl)	% any (<11 g/dl)	N	%	N	%	N
Bandalungwa				1.5	[0.2–5.3]	134	1.0				0.7	51.5	134			22.8	149
Barumbu				2.4	[0.5–6.9]	125	1.7				0.8	57.8	128			19.6	143
Binza Météo				24.8	[17.0–34.0]	109	17.1	1.3	70.6	238	1.7	65.3	118	20.4	339	24.4	131
Binza Ozone				19.1	[12.9–26.7]	136	13.2				1.5	66.2	136			19.0	158
Biyela				46.0	[37.1–55.1]	126	31.7	4.5	64.9	313	2.4	78.6	126	9.5	422	24.7	150
Bumbu	7.4	[4.7–10.9]	299				13.6	3.6	69.2	308				9.3	407		
Gombe				11.5	[6.7–18.0]	139	7.9				3.6	65.5	139			29.6	159
Kalamu I				16.2	[8.4–27.1]	68	11.2				0.0	73.5	68			28.8	73
Kalamu II				2.5	[0.8–5.7]	200	1.7				1.5	56.7	203			19.5	221
Kasa Vubu	2.8	[1.2–5.4]	286				5.1	1.6	55.4	242				10.2	352		
Kikimi				32.8	[24.9–41.6]	131	22.6				2.3	64.9	131			18.5	151
Kimbanseke				36.1	[27.9–44.9]	133	24.9				2.3	75.9	133			22.7	154
Kingabwa	2.6	[1.2–4.9]	345				4.8	1.5	74.3	315				9.8	386		
Kingasani				25.0	[18.3–32.7]	152	17.2				3.3	76.2	151			24.6	175
Kinshasa				0.7	[0.0–4.0]	136	0.5				1.5	61.2	134			30.7	150
Kintambo				11.7	[7.0–18.1]	145	8.1				1.5	68.2	132			24.8	165
Kisenso	11.2	[8.0–15.1]	331				20.5	1.0	69.3	267				8.3	348		
Kokolo^b^	9.3	[6.5–12.9]	353				17.0	0.6	66.7	36				10.3	39		
Lemba				7.7	[3.8–13.7]	130	5.3	1.8	59.4	276	3.1	53.8	130	25.8	357	15.3	150
Limete				17.3	[11.3–24.8]	133	11.9	1.8	69.5	334	3.0	72.2	133	14.3	399	29.1	148
Lingwala				0.7	[0.0–4.1]	135	0.5				0.7	63.0	135			27.3	154
Makala				17.9	[11.8–25.5]	134	12.3				4.5	69.4	134			20.6	155
Maluku II^a^	8.0	[5.0–12.0]	261				14.7	1.2	54.2	260				11.1	342		
Masina I				12.3	[7.3–19.0]	138	8.5				0.7	66.7	138			20.2	163
Masina II				24.8	[17.7–33.0]	133	17.1	2.5	57.6	321	2.3	60.9	133	15.6	458	21.7	161
Matete	3.5	[1.8–6.0]	344				6.4	2.1	74.0	334				6.6	394		
Mont Ngafula I				33.6	[25.7–42.2]	134	23.2				3.7	69.4	134			20.8	154
Mont Ngafula II				35.3	[27.3–44.1]	133	24.4				3.0	68.4	133			19.0	158
Ndjili	6.3	[4.0–9.3]	366				11.5	2.4	61.3	287				16.7	412		
Ngaba				7.5	[3.6–13.3]	134	5.2				1.5	50.7	134			28.8	153
Ngiri Ngiri	1.0	[0.3–2.6]	387	0.8	[0.0–4.2]	124	1.5	1.3	62.4	314	0.8	58.8	131	15.3	428	12.9	140
Police^b^				17.0	[11.1–24.5]	135	11.7				0.7	53.3	135			13.4	164
Selembao	14.1	[10.6–18.2]	347	26.8	[19.9–34.7]	145	23.6	1.9	67.1	319	1.3	65.3	150	15.2	387	19.1	162
Total	6.4	[5.6–7.4]	3319	17.0	[15.7–18.3]	3342	11.7	1.9	65.1	4164	1.9	64.2	3353	13.2	5470	22.3	3841

^a^The HZ of Maluku II, although surveyed in 2009, was excluded from the final risk map since it was chosen to map only HZs of a non-rural character

^b^The HZs Kokolo and Police consist of military and police camps scattered in the city

**Fig. 1:**
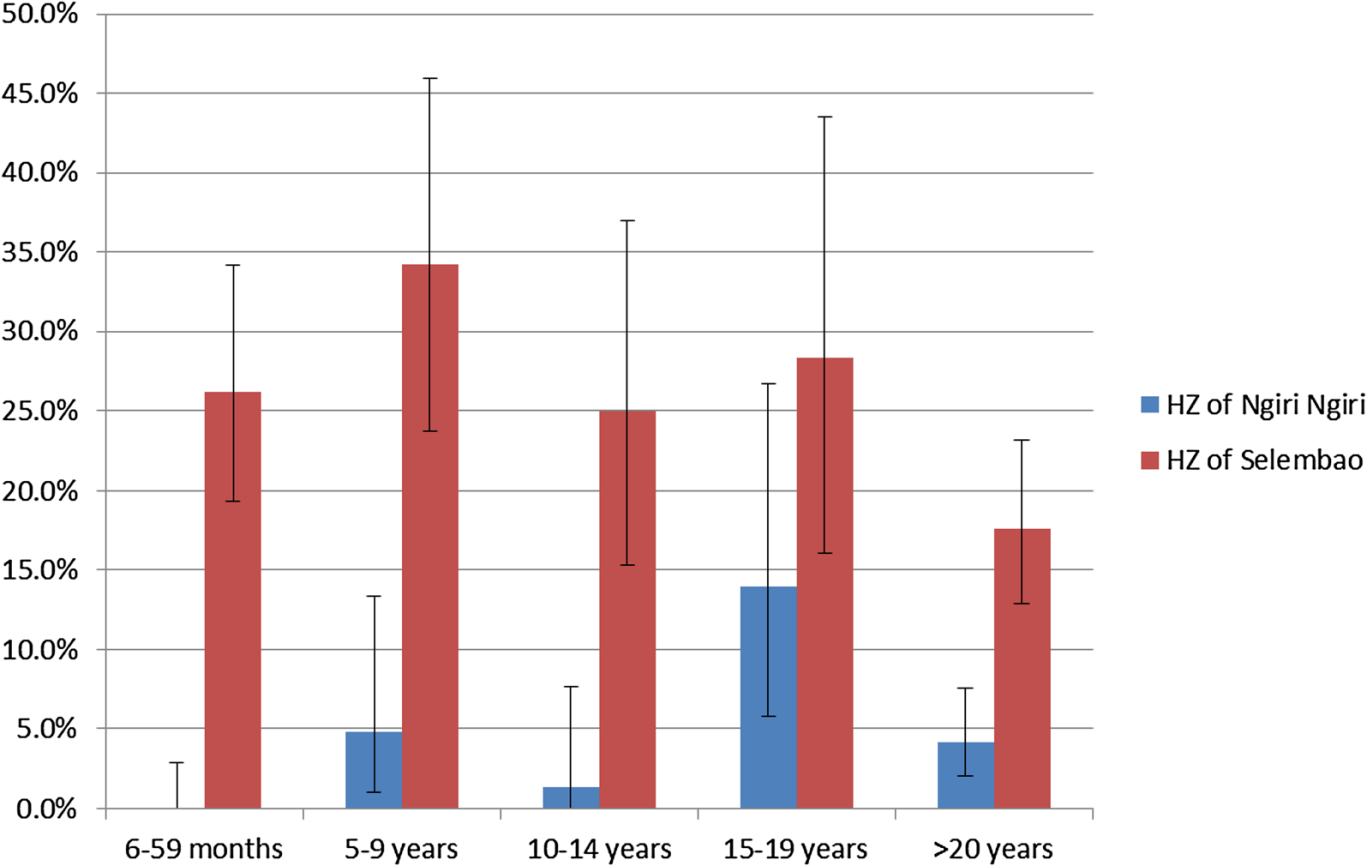
*Plasmodium falciparum* malaria prevalence (RDT positivity) by age group for the health zones of Selembao and Ngiri Ngiri. Bars represent 95 % CI

In the two HZs sampled in both 2009 and 2011, prevalence of malaria in children aged 6–59 months was 1.0 % (0.3–2.6) and 0.8 % (0.0–4.2) in Ngiri Ngiri, and 14.1 % (10.6–18.2) and 26.8 % (19.9–34.7) in Selembao. Age-specific rates (Fig. 1) show that prevalence in Ngiri–Ngiri in 2011 was highest among individuals aged 15–19 years (14.0 %), followed by the groups aged 5–9 years (4.8 %), >20 (4.2 %), 10–14 (1.4 %) and 6–59 months (0.0 %). In Selembao, malaria prevalence was highest among the groups aged 5–9 (34.2 %) and 15–19 (28.3 %) years, followed by those 6–59 months (26.2 %), 10–14 years (25.0 %) and over 20 years (17.6 %). All-ages malaria prevalence was 3.8 % (2.4–5.8) in Ngiri Ngiri and 23.8 % (20.4–27.6) in Selembao.

To prepare the data for mapping, direct standardization was used to make prevalence rates of malaria comparable between surveys, accounting for the two different years and seasons. The standardization was done according to the following formula:

$${P}_{{s}} =\frac{\left( {{{p}}_{{ 1 }} {{n}}_{{ 1 }} \;\bar{p}_{1} \sum {{ n}}_{{ 1 }}\,\,+\,\,{{p}}_{{ 2 }} {{n}}_{{ 2 }} \,\bar{p}_{{2}} \sum {{ n}}_{ 2} } \right)}{\left(\sum {{ n}}_{ 1} + \sum {{ n}}_{ 2}\right)}$$

where, P_s_ is the overall standardized prevalence for surveys 2009 and 2011, p_1_ is the prevalence rate in survey 1 (2009), p_2_ is the prevalence rate in survey 2 (2011), n_1_ is the number of study participants in survey 1, n_2_ is the number of study participants in survey 2, $\bar{p}_{1}$ is the overall prevalence rate for survey 1, $\bar{p}_{2}$ is the overall prevalence rate for survey 2 and ∑ is the total number of study participants (per survey).

### Geographical distribution of *P. falciparum* malaria

**Fig. 2:**
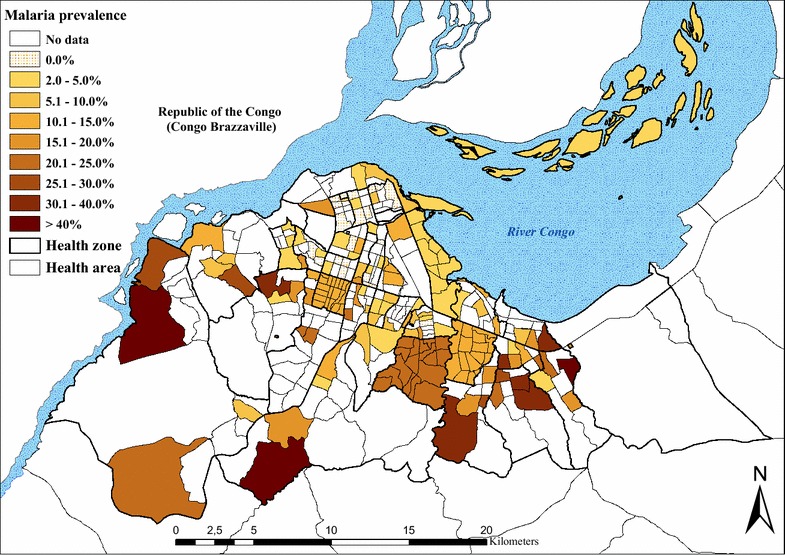
Standardized *Plasmodium falciparum* malaria prevalence in children aged 6–59 months, by health area. The 2009 data for the health zones of Bumbu, Kingabwa, Kisenso, Kokolo and Ndjili were only available at the level of the health zone

**Fig. 3:**
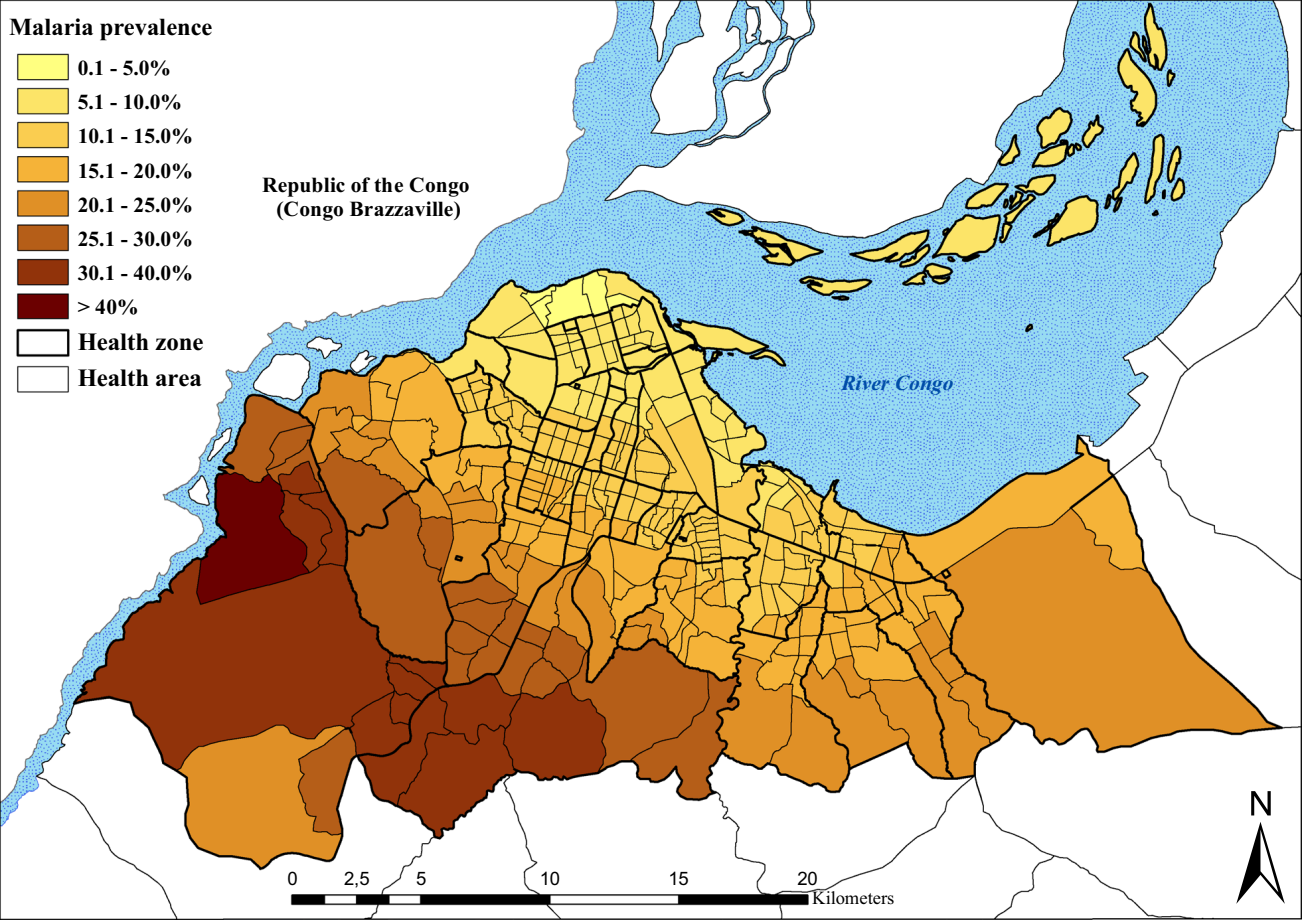
Interpolation results for standardized *Plasmodium falciparum* malaria prevalence in children aged 6–59 months, by health area. Note: Fig. 2 data were used for an inverse distance weighting (IDW) interpolation and then to calculate a mean prevalence value for every health area

Results from the two surveys were used to produce a representative and standardized map of risk for malaria in children aged 6–59 months. Figure 2 shows the spatial distribution of the standardized prevalence rates of *P. falciparum* from the 2009 and 2011 surveys, at the level of the HA. Interpolated standardized prevalence rates are presented in Fig. 3. Based on this risk map, three zones could be approximately defined; low risk in the central north part of the city, where prevalence rates were generally low (≤5 %); intermediate risk in the central southern part of the city, where prevalence rates were between >5 % and ≤30 %; and high risk in the south western and eastern zones, where prevalence rates were higher (>30 %) and, in general, more homogeneously distributed.

### Geographical distribution of anaemia

**Fig. 4:**
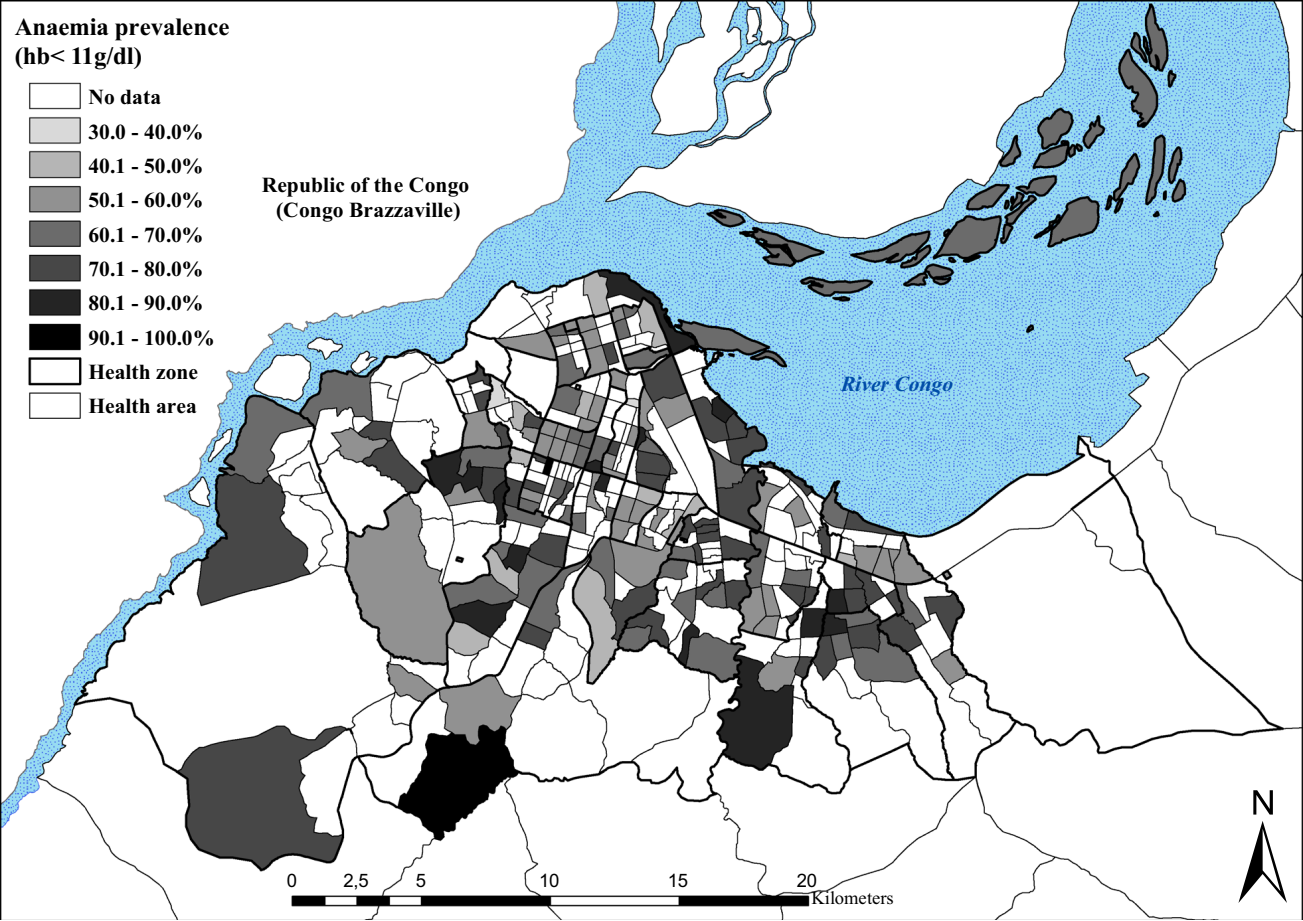
Standardized prevalence of anaemia (Hb < 11 g/dl) in children aged 6–59 months, by health area, surveys 2009 and 2011

**Fig. 5:**
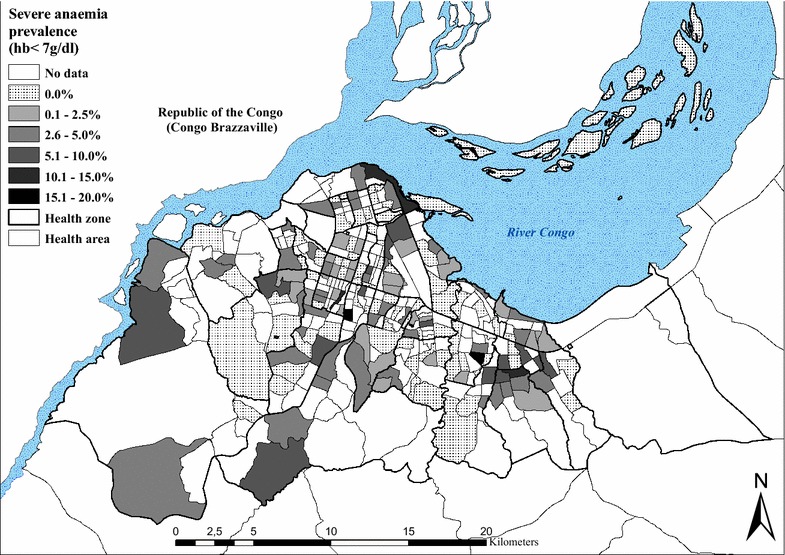
Standardized prevalence of severe anaemia (Hb < 7 g/dl) in children aged 6–59 months, by health area, surveys 2009 and 2011

A total of 4164 and 3353 children aged 6–59 months were tested for anaemia in the 2009 and 2011 surveys, respectively. The mean prevalence of anaemia (Hb < 11 g/dl) was similar between surveys: 65.1 % (63.7–66.6) in 2009 and 64.2 % (62.6–65.9) in 2011. Results also show that the prevalence of moderate (7.0–9.9 g/dl) and severe (<7.0 g/dl) anaemia was 34.2 % and 1.9 % in 2009, and 30.1 % and 1.9 % in 2011 (Table 3). The formula given above was used to standardize the prevalence of anaemia and of severe anaemia. The spatial distribution of the standardized prevalence of anaemia for both surveys is shown in Fig. 4. The risk of anaemia was consistently high across the entire study area, with maximal mean prevalence rates (>70 %) in the HZs of Kingabwa, Matete and Biyela. A map showing the standardized prevalence of severe anaemia is presented in Fig. 5.

### History of fever

The proportion of children aged 6–59 months reporting a history of fever in the 2 weeks preceding the survey was 13.2 % (12.5–14.3) in 2009 and 22.3 % (20.8–23.4) in 2011. On the day of the 2011 survey (data not available for 2009), 3.2 % (106/3348) were febrile (defined as temperature >37.5 °C). The positive predictive value (PPV) of history of fever among children with a positive RDT was 29.7 % (26.3–32.7) in 2011. Health seeking behaviour in case of fever was high in 2011 (data not available for 2009): overall, 91.4 % (770/842) of children sought some type of care. In all, 53.9 % sought modern treatment at home by a family member, whereas 36.1 % were taken to a health facility. Very few (0.5 %) made use of traditional medicine. Private facilities were the most common provider of treatment among those who sought care outside the home, covering 65.4 % of the cases, whereas 22.9 % consulted a public facility and 11.8 % consulted a confessional structure. In case of home treatment, drug outlets represented the principal source of treatment (96.3 %). Unfortunately, only 4.3 % of the anti-malarials purchased were the recommended combination of artesunate-amodiaquine. As a result, only 3.6 % received the recommended treatment at home within the 24 h. In 66.5 % of fever cases, treatment was sought within 24 h regardless of whether treatment was recommended or not.

### Coverage of malaria control measures

Eight months after the 2009 distribution campaign, ITN coverage (measured by the possession of at least one ITN per household) reached 78.7 % (77.4–80.0). In 2011, it was 57.7 % (56.0–59.9). In 2009, coverage ranged from 51.7 % in Biyela (peri-urban) to 92.7 % in Maluku II (peri-urban) (Table 4), with a mean number of 2.0 ITNs per household. In 2011, ITN coverage ranged from 34.4 % in Biyela to 81.8 % in Kinshasa, with a mean number of 1.9 ITNs per household (1.1).

**Table 4:** Malaria control indicators, by health zone

Health zone	Children <5 years having slept under an ITN the night before the survey [95 % CI]				Households that possess at least one ITN [95 % CI]			
	Survey 2009		Survey 2011		Survey 2009		Survey 2011	
	%	N	%	N	%	N	%	N
Bandalungwa			36.2	149			61.0	100
Barumbu			55.9	143			70.7	99
Binza Météo	63.4	331	34.4	131	79.0	200	47.0	100
Binza Ozone			50.0	158			58.0	100
Biyela	30.8	422	17.3	150	51.7	259	34.4	90
Bumbu	81.2	393			91.2	260		
Gombe			57.9	159			68.0	100
Kalamu I			53.4	73			60.0	50
Kalamu II			64.3	221			72.7	150
Kasa Vubu	78.5	302			82.8	263		
Kikimi			27.2	151			37.0	100
Kimbanseke			20.1	154			31.0	100
Kingabwa	68.7	371			66.7	252		
Kingasani			40.8	174			58.0	112
Kinshasa			73.2	149			81.8	99
Kintambo			61.8	165			73.5	102
Kisenso	50.7	341			72.3	242		
Kokolo	72.2	36			92.0	25		
Lemba	66.1	301	36.0	150	83.4	259	60.0	100
Limete	69.0	390	51.4	148	71.8	262	51.0	100
Lingwala			53.2	154			70.0	100
Makala			44.5	155			52.0	100
Maluku II	84.1	292			92.7	260		
Masina I			47.9	163			57.0	100
Masina II	60.7	425	37.3	161	82.1	257	46.0	100
Matete	66.1	387			80.4	260		
Mont Ngafula I			48.1	154			55.0	100
Mont Ngafula II			37.3	158			56.0	100
Ndjili	59.8	381			79.5	258		
Ngaba			67.1	152			74.0	100
Ngiri Ngiri	82.1	418	48.6	140	91.1	259	67.9	106
Police			33.5	164			50.0	102
Selembao	53.3	379	28.4	159	68.1	257	51.0	102
Total	65.0 [63.7–66.3]	5169	45.0 [43.6–46.8]	3835	78.7 [77.4–80.0]	3896	57.7 [56.0–59.9]	2512

The most common reasons for not owning an ITN, as given by households in both the 2009 and 2011 surveys, included not having obtained the ITN during the mass distribution campaign (38.7 and 23.8 %), either because they were absent (26.6 %, 2009) during the campaign or because the stock had been sold out (3.8 and 18.8 %). A high proportion of respondents reported having discarded or destroyed their ITN because of rumours (7.4 and 23.8 %). Other reasons given were heat (2.4 and 10.6 %) and the absence of mosquitoes at home (9.4 % in 2009).

**Fig. 6:**
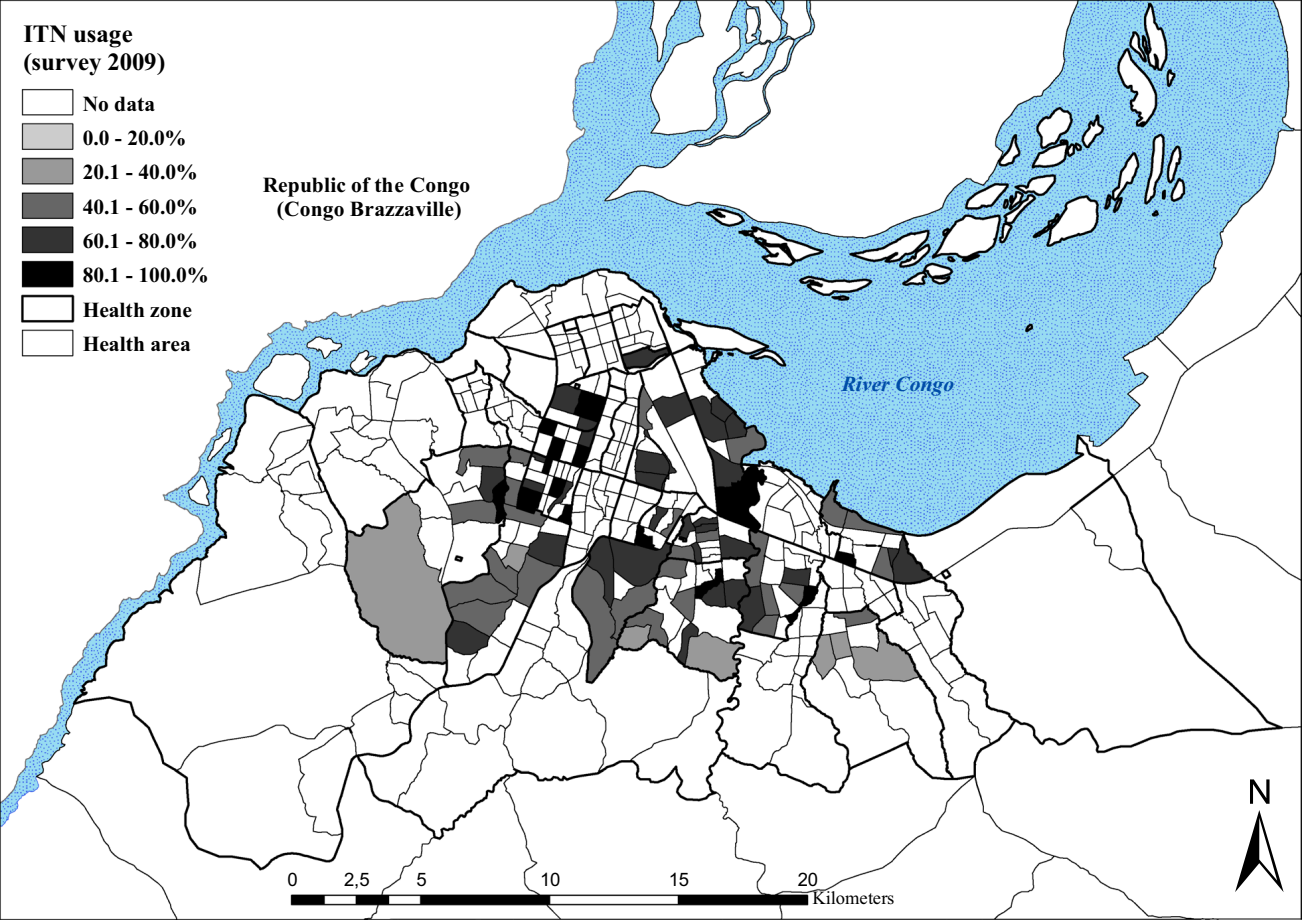
Percentage of children <5 years having slept under an ITN the night before the survey in 2009, by health area

**Fig. 7:**
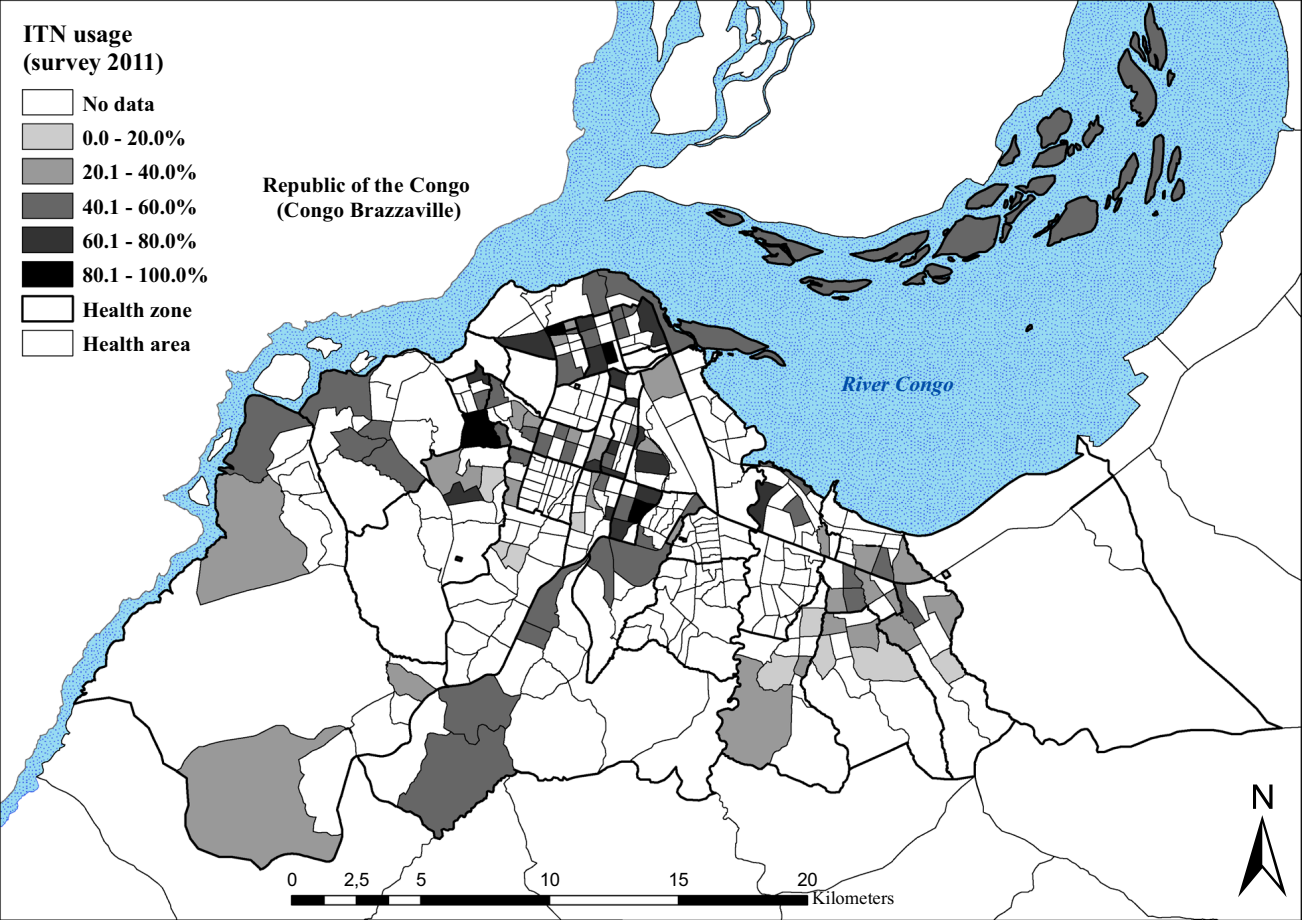
Percentage of children <5 years having slept under an ITN the night before the survey in 2011, by health area

The proportion of respondents who reported that their child slept under an ITN the night before the survey decreased from 65.8 % (63.5–66.0) in 2009 to 45.0 % (43.6–46.8) in 2011. Figures 6 and 7 show the geographical distribution of ITN usage among children under five, geo-referenced and mapped at the level of the HA for both surveys. Use rate decreases progressively towards the periphery in both surveys, with markedly lower use rates (<30 %) in the south-eastern and western health zones of Biyela (30.8 %, 2009; 17.3 %, 2011), Selembao (53.3 % 2009; 28.4 % 2011), Kikimi (27.2 % 2009) and Kimbanseke (20.1 % 2011).

A higher proportion of pregnant women, 83.1 % (77.5–87.7), reported using an ITN in 2009, than in 2011, were reported use decreased to 43.1 % (37.5–48.9). Again, the HZs on the outskirts of the city showed the lowest use rates.

## Discussion

Kinshasa falls within the perennial transmission in the classification of the Mapping Malaria Risk in Africa (MARA) project [15]. This study created the first malaria risk map at the scale of a health area, the lowest level of the health system in DRC. Although the mean endemicity level seems to have declined over the last 30 years in Kinshasa, the results from the 2009 and 2011 cross-sectional surveys show that malaria was still a public health concern [2, 5, 8]. The geographic pattern of endemicity is comparable to that identified in earlier studies done in Kinshasa [2, 5, 8]. Prevalence is clearly highest in the more densely populated and less urbanized districts in the periphery, although marked variations in rates are apparent, even over a few kilometres. These findings are consistent with a recent meta-analysis that used data on the prevalence of malaria parasitaemia to document an analogous situation in other cities in Sub-Saharan Africa [16]. A similar reduction in the annual *P.falciparum* entomological inoculation rates (APfEIR) has been observed in the more urbanized central areas, with a tendency to increase gradually towards the peri-urban areas [17–19]. The relative reduction in the APfEIR in urban areas was also reported in Kinshasa and in Brazzaville [6, 7, 20]. Trape et al. suggested a relationship between levels in transmission in certain districts of Brazzaville and prevalence of malaria reported [20]. The existence of a linear correlation between APfEIR and prevalence was also confirmed by Hay SI et al. [21].

In this study, the overall standardized malaria prevalence was 11.9 % in children 6–59 months, ranging from 0.5 % in the downtown health zones of Kinshasa and Lingwala, to 31.7 % in Biyela, a semi-rural peripheral district extending south east. Results also show that spatial heterogeneity is high in the central and northern urbanized HZs, whereas in the western and south-eastern HZs, more homogeneous levels of high risk can be found (Fig. 2). It is likely that the proximity to productive breeding sites could account for the uneven distribution of malaria risk, together with socio-economic stratification and level of control measures [22–24]. Kazadi et al. observed that initial urbanization might increase levels of malaria transmission through increased human density and the creation of breeding sites favourable to *Anopheles gambiae,* the main local vector [7, 8]. In a second phase, the densification of human habitations reduces potential mosquito breeding sites and hence transmission levels. In Kinshasa, the increase in the density of dwellings in older urban districts has progressively eliminated the last remaining open spaces, contributing to the scarcity of *Anopheles* breeding sites through elimination and pollution. However, exceptions exist, especially where urban agriculture and gardens persist. In particular, the districts extending towards the south-east and west maintain a semi-rural character. Various studies have documented the presence of higher prevalence or transmission rates in areas close to agriculture fields [23, 25–29]. Kinshasa is crossed by rivers from north to south, creating large flood zones where much of the gardening is practised. This characteristic is particularly evident in the large semi-rural areas south west of the *boulevard* Lumumba, encompassing the health zones of Kingasani, Biyela, Kimbanseke and Kikimi. The areas favour *Anopheles sp.* breeding sites and are consistent with the more homogeneous transmission pattern observed in the areas on the outskirts of the city, compared to the more urbanized zones.

Additional factors, such as the use of personal protection against mosquitoes or socio-economic status, should also be considered as important determinants explaining the distribution of disease prevalence. A spatial regression analysis linking malaria prevalence to risk factors for malaria in Kinshasa will be published separately (Ferrari et al. in preparation).

Not surprisingly, the age groups with the highest prevalence, independent from the level of endemicity in both urban and semi-rural areas (Ngiri Ngiri and Selembao), were those aged 5–9 and 15–19. Hence, in Kinshasa, malaria infections seem to occur more frequently late in childhood. This could be in part explained by the age specific ITN usage across age groups, with highest use in younger children in the low endemicity setting (percent of usage in Ngiri Ngiri 49 % compared to 28 % in Selembao) as compared to lower and similar utilization rates among age groups in the high transmission setting (Ferrari et al. in preparation). Higher malaria prevalence rates in older children were also found in school surveys carried out in the 1980s in Kinshasa and in Brazzaville. At that time, the finding was attributed to the increased use of anti-malarials in early childhood [20, 30].

A concerted effort to scale-up ITN coverage through a free distribution in Kinshasa led to an ITN ownership rate of 78.7 % of households in 2009. This represented a 395 % increase in household possession of ≥1 ITN over the 2007 estimate of only 15.9 % [9]. However, 24 months after the distribution campaign, ITN ownership had decreased to 57.7 % of households. Clearly, this points to the need for stronger programmes for routine ITN distribution as it occurs in most endemic settings, in addition to the campaigns [31].

The prevalence of anaemia was high in 2009 (65.1 % in children 6–59 months) and in 2011 (64.2 %). This is consistent with the 69.2 % prevalence reported by the DHS 2007 [9]. Furthermore, the distribution of anaemia across Kinshasa was highly heterogeneous as shown in Fig. 4, and the absence of a spatial trend seems in favour of the role of additional factors other than malaria in the aetiopathogenesis of this condition. Multiple factors account for anaemia and their contributions can vary according to the setting [32]. In Kinshasa, 23 % and 9 % of children suffer from chronic and severe forms of malnutrition [9] and sickle cell anaemia is widespread [33].

The maps we present for different variables reflect survey results from two distinct time periods and seasons. Since malaria transmission is neither constant throughout the year nor between years, this has likely introduced some mistakes. To account for these differences, we tried standardizing prevalence rates, but that is an imperfect means of accounting for such differences. Moreover, surveys were based on detection of cases of uncomplicated malaria and, therefore, it is not possible to draw strong conclusions about the prevalence of anaemia, which is more often related to severe malaria.

## Conclusions

This study provides the first comprehensive risk map of malaria at the level of the health areas in Kinshasa, a mega-city in a highly endemic malarious zone. Overall malaria prevalence has undoubtedly decreased over the last 30 years, but it is impossible to quantify the effect given the lack of representative historical data. As expected, prevalence rates were lower in the central urban districts compared to the more peripheral and more rural districts [8]. The penetration of malaria control measures, especially ITNs, remains insufficient and is less successful in less developed and less accessible HZs on the outskirts of the city. Hopefully, this gap can be closed in the years to come with renewed efforts by the National Malaria Control Programme and its partners. Despite the methodological limitations, the risk map provides a good baseline assessment against which to assess the effect of future control efforts.

## Abbreviations

ALu: artemether-lumefantrine
AS-AQ: artesunate-amodiaquine
ADB: Asian Development Bank
GIS: geographic information system
GPS: global positioning system
GF: global fund
HA: health area
HZ: health zone
ITN: insecticide-treated net
KSPH: Kinshasa School of Public Health
NMCP: National Malaria Control Programme
MoH: Ministry of Health
PSI: Population Services International
RDT: rapid diagnostic test
UNICEF: United Nations Children’s Fund
USAID: United States Agency for International Development
JICA: Japanese International Cooperation
